# Effect of Delayed Light-Curing Through a Zirconia Disc on Microhardness and Fracture Toughness of Two Types of Dual-Cure Cement

**Published:** 2018-11

**Authors:** Pouran Samimi, Sara Kaveh, Maryam Khoroushi

**Affiliations:** 1 Associate Professor, Torabinejad Dental Research Center, Department of Operative Dentistry, School of Dentistry, Isfahan University of Medical Sciences, Isfahan, Iran; 2 Assistant Professor, Department of Operative Dentistry, School of Dentistry, Semnan University of Medical Sciences, Semnan, Iran; 3 Professor, Torabinejad Dental Research Center, Department of Operative Dentistry, School of Dentistry, Isfahan University of Medical Sciences, Isfahan, Iran

**Keywords:** Light-Curing of Dental Adhesives, Hardness, Dental Stress Analysis, Material Testing, Resin Cements, Zirconium Oxide

## Abstract

**Objectives:**

Photopolymerization immediately sets dual-cure cements and prevents the continuation of chemical polymerization. Delayed light-curing allows the chemical process to continue up to the point before starting irradiation; however, there is a controversy in this respect. The present study evaluates the effect of delayed light-curing through a zirconia disc on the microhardness and fracture toughness (K_IC_) of two types of dual-cure cement.

**Materials and Methods:**

Samples measuring 25×5×3 mm^3^ were prepared for fracture toughness test, and discs measuring 5 mm in diameter and 3 mm in thickness were prepared for microhardness test using Bifix and BisCem cements. Light-curing protocols were as follows: immediate light-curing (group A), a 2-minute delay (group B), a 5-minute delay (group C), direct irradiation (group D), and no irradiation (group E). In groups A to C, light-curing was carried out through a zirconia disc. Data were analyzed by two-way and one-way analysis of variance (ANOVA), post-hoc Tukey's test, and Kruskal-Wallis test at 95% confidence interval.

**Results:**

There was a significant difference in the microhardness of the cements (P=0.00). Delayed light-curing had no effect on microhardness (P=0.080). The microhardness of BisCem in group E was significantly lower than that in group D (P=0.015). The fracture toughness of Bifix in groups B and C was significantly different than that in group E and BisCem groups.

**Conclusions:**

Under the limitations of our study, delayed light-curing had different effects on microhardness and fracture toughness. Differences in light-curing protocols resulted in different effects based on the cement type. Light-curing is recommended to achieve optimal mechanical properties.

## INTRODUCTION

Since their introduction in the 1970s, the formulation of resin cements has been hugely improved [1]. Self-cure materials do not have set-on-command capability, while light-cure ones cannot ensure proper polymerization in areas with limited access for the curing light [2]. To overcome the limitations of these two modalities, dual-cure resin cements have been introduced and are extensively used in modern adhesive restorative dentistry because of their noticeable advantages [3].

Although the concept of using these dual-curing materials appears to be appealing, several issues have been raised in the literature. It has been hypothesized that light activation may negatively affect the self-curing mechanism, preventing the achievement of maximum mechanical properties [4–8]. The rationale is that immediate light activation leads to rapid formation of a cross-linked polymer, resulting in entrapment of reactive species, including initiators and activators, within the network [9]. This is clinically important because light-curing is recommended in most cases. Photoactivation of dual-cure cements can be delayed as much as possible so that the overall concentration of free radicals would increase, improving the conversion rate and finally giving rise to better mechanical properties [9]. Under such conditions, the self-curing process continues up to a point that it would not interfere with the light-curing process [1]. Delayed light activation protocols have been evaluated in various studies using different methods to determine their effect on the physical and mechanical properties of dual-cure cements [10,11]. Some studies on the effect of the curing protocol have evaluated the degree of conversion of polymers [12–15]. However, it seems that the mechanical properties of a cement should be favorable in order to support the restoration and to increase the resistance against functional stresses over time. Of all the different properties, the fracture toughness (K_IC_) of a cement is a valuable mechanical property that can be used to predict the clinical performance of the cement since one of the primary causes of treatment failure is cement fracture [16]. Cement fracture and microleakage have been reported to be the main etiologies for the failure of cemented restorations, which might be attributed to poor cementation techniques, the inappropriate design of the prosthesis, poor fitting of the casting, malocclusion, unretentive preparation, or a weak cement [16]. Fracture toughness is an inherent property that indicates the ability of a material to resist crack propagation [17,18]. To date, the effect of delayed photoactivation on the fracture toughness of dental cements has not been evaluated.

Another important mechanical property of dental cements is the surface microhardness, which is useful for indirect evaluation of the conversion of composite resin matrix [19]. To date, the time intervals set for evaluation of the effect of delayed photoactivation on the microhardness of dental cements have been beyond the clinically acceptable levels [13]. Since saving time is important in the clinic, one of the aims of the present study was to evaluate and compare shorter time intervals.

The aim of this study was to evaluate the effect of immediate and delayed light-curing (at two time intervals of 2 and 5 minutes) on the microhardness and fracture toughness of two commercially available self-adhesive resin cements under zirconia restorations. The tested hypotheses were: 1) Delayed light-curing will affect the microhardness of the cements; 2) Delayed light-curing will affect the fracture toughness of the cements; 3) The zirconia disc will not affect the microhardness or the fracture toughness of the cements.

## MATERIALS AND METHODS

In the present in-vitro study, the effect of delayed light-curing through a zirconia disc was evaluated on the mechanical properties of two types of resin cement. Microhardness and fracture toughness tests were carried out. Two dual-cure self-adhesive cements were used. Table 1 presents the particulars of the evaluated cements.

**Table 1.** The particulars of the tested cements

** Cement **	** Composition **	** Curing time **
Bifix SE (VOCO GmbH, Cuxhaven, Germany)	Aliphatic (UDMA), aromatic (Bis-GMA) and acid methacrylate, benzoyl peroxide (initiator), amines (cat) and BHT (stabilizer).	20 seconds
BisCem (Bisco Inc., Schaumburg, IL, USA)	Base: Bis-GMA, uncured dimethacrylate monomer, glass filler. Catalyst: Phosphate acidic monomer, glass filler.	20–30 seconds

Bis-GMA=Bisphenol A-Glycidyl Methacrylate, UDMA= Urethane Dimethacrylate, BHT=Butylated Hydroxytoluene

### Zirconia disc fabrication:

Bar-shaped zirconia blocks (30×10×1.5 mm^3^; A1 shade; Amann Girrbach AG, Koblach, Austria) were prepared using a computer-aided design/computer-aided manufacturing (CAD/CAM) system (Amann Girrbach AG, Koblach, Austria). The fabricated blocks were thoroughly polished, and their thicknesses were controlled using a digital micrometer (QLR Electronic Micrometer-IP54, Fowler High Precision Inc., Massachusetts, USA).

### Vickers microhardness test:

A microhardness tester (Leitz Wetzlar Metallux 3, Spectrographic Ltd., Guiseley, Leeds, UK) was used for Vickers microhardness test. Disc-shaped dark plexiglass molds with the diameter of 5 mm and the thickness of 3 mm were used to prepare 10 resin cement specimens in each group. A laser cutting machine (Shenzhen Herolaser Equipment Co., Ltd., Shenzhen, Guangdong, China) was used to prepare the samples. The following formula was used to calculate the sample size:

$$\frac{{\left(Z_{1-\frac{\alpha }{2}}+Z_{1-\beta }\right)}^{2}\left(\delta_{1}^{2}+\delta_{2}^{2}\right)}{d^{2}}$$

in which α is the alpha error (5%), β is the beta error (20%), δ is the variance in the two groups (0.05), and d is the minimum effect size (4.5 N/mm2). With 10 samples in each group, there was an 80% odds of a minimum difference of d=4.5 N/mm2 between the means of the groups at a significance level of α=5%.

The resin cements were retrieved from the refrigerator one hour before the tests [8]. The automixed cements were injected into the cavities using a special disposable needle from the cement kit. A Mylar band (TDV Dental Ltda., Pomerode, Santa Catarina, Brazil) was placed on the cement, and the zirconia disc was placed on the band. Hand-made jigs were fabricated using self-curing acrylic resin (AcroPars 200; Marlic Medical Industries Co., Tehran, Iran) to ensure an identical position between the zirconia discs and the tip of the conductor of the light-curing unit in all the samples.

The cement samples were divided into the following groups based on the light-curing protocol:

Group A: Light-curing immediately after being placed in the mold
Group B: Light-curing with a 2-minute delay
Group C: Light-curing with a 5-minute delay
Group D: Direct light-curing (without zirconia disc)
Group E: No light-curing (control)

In group E, there was no zirconia disc, and the cement was protected against the light during polymerization. Based on the recommendations of the manufacturers, the samples in groups A, B, and C were light-cured for 20 seconds at a light intensity of 800 mW/cm^2^ using a VALO^®^ light-curing unit (VALO^®^, Ultradent Products Inc., South Jordan, UT, USA). After light-curing of every 5 samples, the light intensity of the unit was checked using a radiometer (Demetron Light-Emitting Diode (LED) Radiometer Model 100, SDS/Kerr, Danbury, Connecticut, USA). During the light-curing process, the tip of the light conductor touched the surface of the zirconia block [8]. Immediately after fabrication, the samples were placed in a black box with no light penetration at 100% relative humidity. The samples were then placed in an incubator (01154, Behdad Digital Incubator, Tehran, Iran) for one week [8]. The microhardness (Kg/mm^2^) of each sample was determined at three points, and the mean of the three values was reported as the microhardness of each sample. The points were 0.5 mm apart. A load of 100g was applied with a dwell time of 10 seconds [9].

### Fracture toughness test:

**Fig. 1.**
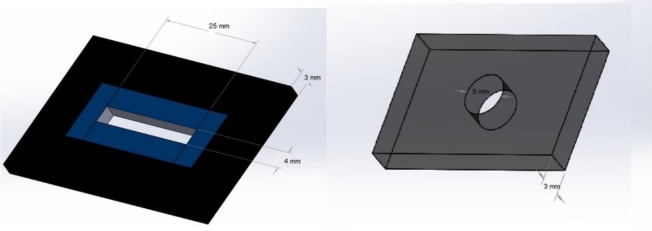
Schematic representation of the cement mold used in fracture toughness (K_IC_) and microhardness tests

**Fig. 2.**
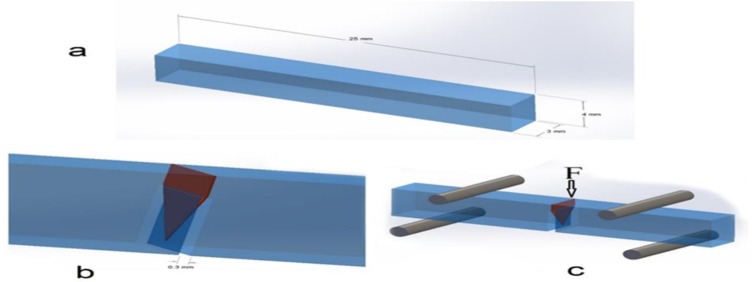
Schematic representation of a cement sample under fracture toughness (K_IC_) testing with four-point deflection

The Chevron Notched Beam (CNB) technique was used for fracture toughness test [20]. There were five samples in each group. Due to the lack of similar studies, the sample size was determined based on the recommendations of ISO 24370:2005(E) [7]. The samples were prepared using each cement, and light-curing procedures were carried out as described above for the microhardness test. Dark plexiglass split-molds were prepared to make bar-shaped specimens (25×4×3 mm^3^). An arrow-shaped notch was formed on the sample in a non-stop cutting machine (Dentarapid, Krupp Dental, 759DRZ, Hilzingen, Germany) using a diamond disc (Dumont^®^ Sintered Diamond Discs, Dumont-Instruments & Co NV, Vorst, Belgium), extending 3.5 mm into the sample. The diameter of the notch was less than 3 mm. Schematic representation of a sample and its position in the universal testing machine (K-21046; Walter+Bai AG, Löhningen, Switzerland) are presented in Figures 1 and 2. The dimensions of the specimens conformed to ISO 24370(E) standards [21]. The samples underwent a four-point deflection test [20].

The fracture toughness (MPa/mm^2^) was calculated using the fracture load (N) and the minimum coefficient of stress [7]. A proper test machine was used for the test (a machine capable of applying force at a constant crosshead speed). The test machine conformed to ISO 7500-1:2004 Class 1 [21], capable of calculating fracture toughness with 1% accuracy. Next, the fracture toughness was calculated using the following formula:

$$K_{I,\mathrm{CNB}}=\frac{F\left(S_{0}-S_{i}\right)}{BW^{\frac{3}{2}}}\times \frac{Y_{\min}}{\sqrt{1000}}$$

where W is the width of the specimen (4 mm), B is the thickness of the specimen (3 mm), *S*_0_ is 2 mm, *S_i_* is 1 mm, *Y_min_* is 4.09, and F is the maximum force necessary to fracture the sample (F_max_).

### Statistical analyses:

Kolmogorov-Smirnov test was used to evaluate the normal distribution of the data. One-way and two-way analyses of variance (ANOVA) were used to analyze the results of Vickers microhardness tests. Also, Bonferroni test was used for pairwise comparisons.

## RESULTS

### Vickers microhardness test:

**Fig. 3.**
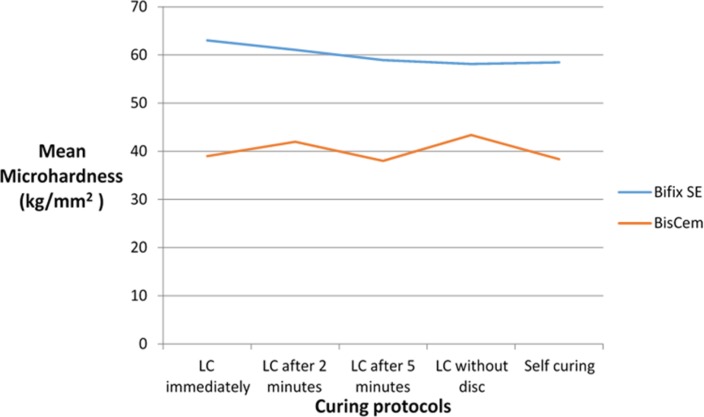
Mean microhardness (Kg/mm^2^) of the two cement types according to the curing protocols, LC=light-curing

**Fig. 4.**
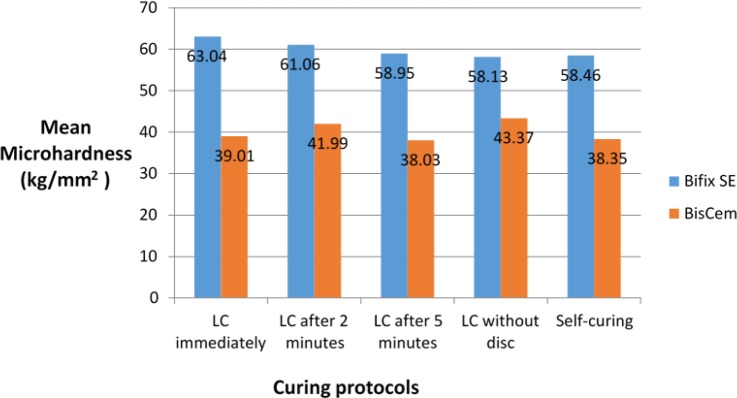
Mean microhardness (Kg/mm^2^) of the two cement types according to the curing protocols, LC=light-curing

The mean and standard deviation (SD) of microhardness for the two cements in different light-curing protocols are presented in Table 2 and Figures 3 and 4. The cement type had a significant effect on microhardness (F(1,90)=474.8; P<0.001; R^2^=0.841) such that the microhardness of Bifix was 8.19 Kg/mm^2^ more than that of BisCem. Moreover, there was a strong correlation between the cement type and different light-curing protocols (F(1,90)=2.74, P=0.033, R^2^=0.109). The mean microhardness of Bifix was more than that of BisCem with all the light-curing protocols (P<0.001).

**Table 2.** Mean microhardness (Kg/mm ^2^ ), standard deviations (SD), 95% confidence intervals (CI), and minimum and maximum values in the resin cements under study

** Cement **	** Curing protocol **	** Mean **	** SD **	** 95% CI **		** Min **	** Max **

				** Lower **	** Upper **		
** Bifix SE **	LC immediately	63.04	4.01	60.16	65.90	57.35	70.05
	LC after 2 minutes	61.06	8.19	55.19	66.91	45.90	69.05
	LC after 5 minutes	58.95	4.01	56.08	61.81	53.45	64.90
	LC without disc	58.13	5.09	54.48	61.76	49.60	65.85
	Self-curing	58.46	3.91	55.66	61.25	52.60	63.70
** BisCem **	LC immediately	39.01	4.57	35.74	42.27	29.25	44.15
	LC after 2 minutes	41.99	3.49	39.49	44.49	34.85	46.65
	LC after 5 minutes	38.03	2.80	36.02	40.03	32.85	41.30
	LC without disc	43.37	3.12	41.13	45.60	39.25	48.80
	Self-curing	38.35	3.84	35.59	41.09	34.65	46.70

LC=light-curing

### Fracture toughness test:

**Fig. 5.**
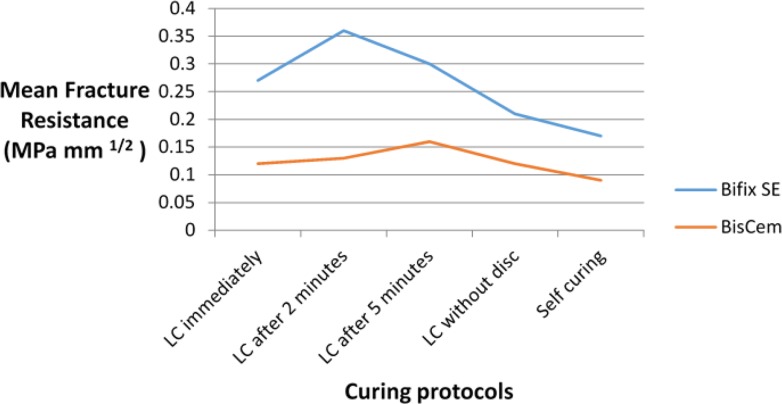
Mean fracture resistance (K_CI_; MPa/mm^2^) of the two cement types according to the curing protocols, LC=light-curing

**Fig. 6.**
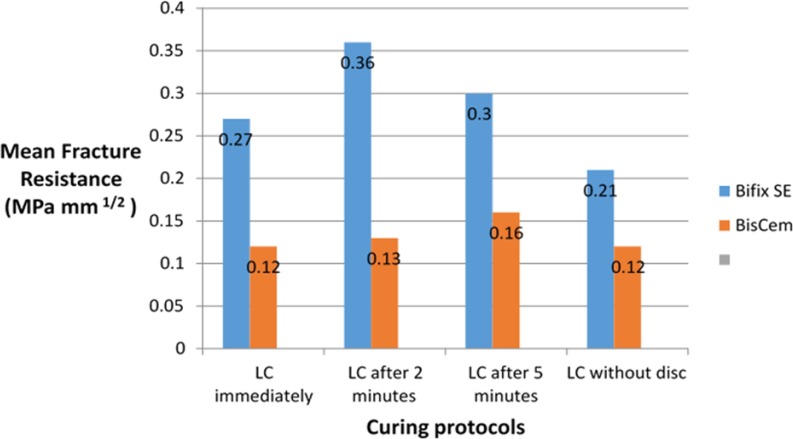
Mean fracture resistance (K_CI_; MPa/mm^2^) of the two cement types according to the curing protocols, LC=light-curing

The mean and SD of fracture toughness for the two cements in different light-curing protocols are presented in Table 3 and Figures 5 and 6. The cement type (F(1,38)=474.8, P<0.001, R^2^=0.841) and different light-curing protocols (F(4,38)=3.55, P=0.015, R^2^=0.272) had a significant effect on fracture toughness. But the correlation between the cement type and different light-curing protocols was not significant with regard to the fracture toughness.

**Table 3.** Mean fracture toughness (K _IC_ , MPa/mm ^2^ ), standard deviation (SD), 95% confidence intervals (CI), and minimum and maximum values in the resin cements under study

** Cement **	** Curing protocol **	** Mean **	** SD **	** 95% CI **		** Min **	** Max **

				** Lower **	** Upper **		
** Bifix SE **	LC immediately	0.27	0.12	0.12	0.42	0.10	0.38
	LC after 2 minutes	0.36	0.13	0.20	0.53	0.21	0.51
	LC after 5 minutes	0.30	0.05	0.24	0.36	0.25	0.35
	LC without disc	0.21	0.10	0.08	0.34	0.10	0.37
	Self-curing	0.17	0.07	0.08	0.25	0.10	0.24
** BisCem **	LC immediately	0.12	0.04	0.07	0.18	0.09	0.20
	LC after 2 minutes	0.13	0.04	0.09	0.18	0.07	0.16
	LC after 5 minutes	0.16	0.08	0.06	0.26	0.05	0.26
	LC without disc	0.12	0.05	0.00	0.24	0.07	0.17
	Self-curing	0.09	0.04	0.05	0.14	0.05	0.51

LC=light-curing

With Bifix cement, the mean fracture toughness was approximately 14.0 MPa/mm^2^ more than that of BisCem cement. In addition, the fracture toughness was significantly higher with the “2-minute delay” light-curing protocol compared to no light-curing (P=0.018).

## DISCUSSION

This study evaluated the effect of different light- curing protocols on the microhardness and fracture toughness of two resin cements. Based on the results, delayed light-curing did not increase the microhardness. In terms of the fracture toughness, delayed light-curing increased the K_IC_ of Bifix but the results were not significant with BisCem.

### Effect of curing protocol on microhardness:

There was a significant difference between the microhardness of the two tested cements, which is in agreement with the results of similar studies. Ramos et al [22] studied the microhardness of two cements (Panavia F and Rely X U100) and showed significant differences. Cekic-Nagas et al [23] evaluated Panavia F, Bifix-QM, Nexus, Duolink, and Rely X Unicem cements and reported that the type of cement was an effective factor in the microhardness.

The main factors determining the mechanical properties of resin-based materials are the degree of conversion, cross-linking density, and filler content [24–26]. Since different resin cements have different compositions and various catalyst systems, polymerization kinetics, the extent of polymerization, and consequently the mechanical properties of the cement change considerably in various curing protocols [27]. For example, the diametrical tensile strength (DTS) of Panavia F cement decreases in the absence of light, which is not observed with Rely X ARC and Enforce cements. Contrary to Panavia F, the other two cements are bisphenol A-glycidyl methacrylate (Bis-GMA)-based, and use of this stiff monomer in resin-based cements increases the cross-linking density in the polymer. In addition, deficiency in chemical curing can result in a higher density of unreacted double bonds, lower hardness, and higher solubility of cements, which affect the chemical stability in the oral cavity [11].

In the present study, delayed light activation for 2 and 5 minutes did not lead to a significant increase in the microhardness of the two cements, which is consistent with the results of other related studies. In a recent study on Rely X ARC resin cement with delayed light activation for 0, 1, and, 2 minutes, light activation was carried out with four different light intensities, and the degree of conversion and microhardness were measured; a 1-minute delay improved the degree of conversion and microhardness of the cement but a 2-minute delay did not [28]. Considering the close correlation between microhardness and degree of conversion, one can rely on the results of the work by Pereira et al [27]; however, in the 10-minute delay group, the self-curing mechanism progressed such that the cement reached the vitrification phase, that is, because of an increase in cross-linking density, the viscosity of the cement increases to the level that limits the activity of reactive terminals [27]. Group D of BisCem cement exhibited a higher microhardness compared to group E, demonstrating the importance of light-curing with some self-adhesive resin cements [11,12, 29]. In a study by Faria-e-Silva et al [9], all the three cements exhibited a lower degree of conversion in the absence of light, giving rise to a weaker polymer [30–32]. It should be pointed out that the Bisco Company (BisCem cement) claims that it is possible to apply the cement in a self-curing manner without the application of light [32]; however, it has been mentioned in the manufacturer’s instructions that if the cement is used in a self-curing manner, it is advisable to slightly light-cure the margins or use limited light-curing for 20–30 seconds [32]. Based on the claims made by the VOCO Company (Bifix cement) [33] and given the possible limitations in the clinic, it might not be possible to provide ideal conditions, which indicates the importance of light-curing for achieving maximum cement properties.

### Effect of curing protocol on fracture toughness:

Compared to studies on microhardness, there are only limited studies available on the fracture toughness of resin cements. No study has evaluated the effect of delayed light-curing on the K_IC_ of cements. There is no consensus on how to carry out the test, which makes it difficult to compare the results.

Another important factor is the diluent monomer like triethylene glycol dimethacrylate (TEGDMA), which might lead to lower water sorption and consequently higher fracture toughness [34]. The volume fraction of fillers and the filler type/size are also important; it has been shown that a filler content up to 57% will boost the fracture toughness, while higher concentrations will have a reverse effect, which is probably due to increased accumulation of flaws, voids, porosities, and filler agglomerates as a consequence of a higher viscosity [35].

In the present study, the fracture toughness was significantly higher with Bifix compared to BisCem, which might be related to the filler content of the cements (70 wt% versus 50 wt%). It has been reported that preventing crack propagation by diverting the crack propagation path results in an increase in fracture energy, leading to increased fracture toughness [36]. It should be pointed out that very high filler content is one of the factors that can make the material susceptible to fracture because, under these conditions, the fillers function as stress concentration areas and result in fracture propagation and a decrease in fracture toughness [37].

In the present study, the fracture toughness of the groups with delayed light-curing was significantly higher with Bifix cement, and the results of a 2-minute delay were better than those of a 5-minute delay. A 2-minute delay has not been evaluated extensively in other studies. Since this short delay had similar effects to the 5-minute delay, both are recommended, but further studies are necessary. In addition, the fracture toughness in groups A and D of Bifix cement was significantly higher than that of group E of BisCem cement, possibly indicating the importance of light-curing to achieve maximum properties.

### Effect of zirconia disc on the evaluated mechanical properties:

The other parameter assessed in the present study was the effect of a zirconia disc on the mechanical properties of the cements. It seems that despite the well-reported attenuation of light intensity by indirect restorations, the 1.5-mm-thick zirconia disc did not affect any of the mechanical features tested. These results are consistent with those reported previously. A study by Shiomuki et al [38] on the Vickers microhardness of Panavia F dual-cure resin cement through a zirconia disc showed no significant differences in microhardness between the areas exposed to direct light and those away from light.

Since a number of factors affect the efficacy of polymerization of dual-cure cements, including duration of irradiation [39], light intensity [40], conductance of light [41,42], and the color and thickness of ceramic [43,44], in many cases, the negative effect of one factor can be compensated by other factors. Flury et al [29] reported that decreased irradiance in combination with prolonged light-curing times did not jeopardize the micromechanical properties of resin cements even through 1.5-mm-thick discs. On the other hand, it has been hypothesized that direct light-curing of a cement has a negative effect on the cement properties due to the relatively high irradiance, that is, the higher concentration of radicals results in premature termination of polymerization and causes deficiencies in the formation of the polymer network [45–47]. Therefore, the negative effect of restoration on irradiance has been considered a positive effect. For example, in a study by Ozturk et al [47], the Vickers microhardness and elastic modulus of cements were significantly higher through a ceramic disc measuring 0.75 mm in thickness compared to direct light-curing. In the above study, when light-curing was carried out through discs measuring 3 mm in thickness, most resin cements showed a significant reduction in the parameters in question. Therefore, in such cases, an increase in the light-curing time has been recommended to compensate for the deficiency [30]. The shade of the veneering material also affects the amount of the transmitted light and consequently the microhardness of the cement. In the present study, the A1 shade was used. Barghi and McAlister [48] reported that high-chroma restorative materials decrease the microhardness of the underlying cement. However, it has been reported that the material thickness has a greater effect on light transmission compared to the shade of the material [49]. Since the polymerization efficiency of dual-cure resins is affected by numerous factors such as irradiation duration, light intensity, light transmission, ceramic thickness, and ceramic shade, it is practical to compensate for the effect of one factor by modifying the others as it has been demonstrated that an extended period of light-curing is a helpful way to improve the degree of conversion of the cement through glass ceramics [50].

Further research on the effect of zirconia disc seems necessary. In addition, further studies on the chemical compositions of cements are suggested in order to more accurately evaluate the effect of each component of the cement on their curing mechanism and mechanical properties. Furthermore, it is useful to evaluate the effect of different restorations on delayed light-curing and curing mechanisms of resin cements. It is recommended that delayed light-curing protocols be further evaluated using dual-cure resin cements.

## CONCLUSION

Under the limitations of the present study, it can be concluded that:

1. The fracture toughness and microhardness of the studied resin cements were different.
2. Delayed light-curing had different effects on the microhardness and fracture toughness of the two cements.
3. Light-curing is strongly recommended since self-curing cannot provide acceptable mechanical properties for the clinical use.
4. Light-curing through an A1-shade zirconia disc with a thickness of 1.5 mm had no effect on the fracture toughness and microhardness of the studied cements.
